# Title Medico-Legal Preparedness in Dental Education: Training Gaps, Defensive Practice and Curriculum Implications Among Romanian Dental Students and Dentists

**DOI:** 10.3390/dj14090579

**Published:** 2026-09-09

**Authors:** Larisa Pătru, Ciprian Laurențiu Pătru, Elena Cristina Andrei, Oana Andreea Diaconu, Maria Cristina Comănescu, Daniela-Maria Micu, Virginia Maria Rădulescu

**Affiliations:** 1 Department 9, Faculty of Medicine, University of Medicine and Pharmacy of Craiova, 200349 Craiova, Romania; larisa.patru@umfcv.ro; 2 Department 8 Mother and Child, Faculty of Medicine, University of Medicine and Pharmacy of Craiova, 200349 Craiova, Romania; 3 Department of Histology, Faculty of Dentistry, University of Medicine and Pharmacy of Craiova, 200349 Craiova, Romania; elena.andrei@umfcv.ro; 4 Department of Endodontics, Faculty of Dentistry, University of Medicine and Pharmacy of Craiova, 200349 Craiova, Romania; 5 Department of Anatomy, Faculty of Medicine, University of Medicine and Pharmacy of Craiova, 200349 Craiova, Romania; maria.comanescu@umfcv.ro; 6 Department of Foreign Languages, University of Medicine and Pharmacy of Craiova, 200349 Craiova, Romania; daniela.micu@umfcv.ro; 7 Department of Medical Informatics and Biostatistics, Faculty of Medicine, University of Medicine and Pharmacy of Craiova, 200349 Craiova, Romania; virginia.radulescu@umfcv.ro

**Keywords:** dental malpractice, defensive medicine, medico-legal preparedness, legal awareness, dental education, dental students, cross-sectional study, Romania

## Abstract

**Background/Objectives:** Medico-legal preparedness is relevant throughout dental education because awareness develops before independent clinical responsibility is assumed. This study examined malpractice awareness, self-perceived preparedness, legal awareness and defensive-practice tendencies among Romanian dental students, residents and dentists. **Methods:** A cross-sectional survey was conducted between May and June 2026 among 176 participants from the Faculty of Dentistry and its associated professional community in Craiova, Romania. The author-developed questionnaire was completed online (n = 134) or on paper at a professional conference (n = 42). Associations were assessed using non-parametric tests, ordinal logistic regression and robust linear regression. A sensitivity analysis excluded early-year students. **Results:** Respondents demonstrated moderate legal awareness (mean 3.29 of 5), whilst only 8.0% felt well or very well prepared to manage a malpractice-related situation. In the main model, previous legal training was associated with greater preparedness (adjusted odds ratio 2.52, 95% CI 1.03–6.15), as were resident status and professional clinical experience. In the clinically exposed subgroup (n = 68), practising dentists reported greater preparedness than clinical-year students, whereas familiarity with malpractice and paper-based participation were associated with stronger defensive-practice tendencies. **Conclusions:** Awareness of medico-legal issues is present across dental education, but perceived preparedness remains limited. The findings support longitudinal, applied medico-legal education and require cautious interpretation because early-year and clinically experienced respondents evaluated partly different aspects of the same phenomenon.

## 1. Introduction

Patient safety remains a central priority for contemporary healthcare systems. The World Health Organisation (WHO) Global Patient Safety Action Plan 2021–2030 identifies the prevention of avoidable harm as a strategic objective and calls for strengthening safety across clinical practice, healthcare governance, professional education and health policy [1]. Malpractice is not itself a direct measure of patient safety: it is a legal attribution that may follow an alleged breach of professional duty and demonstrable harm. Nevertheless, malpractice complaints and claims can reveal recurrent problems in communication, consent, documentation, clinical processes and organisational safeguards. They may therefore provide an indirect, incomplete indicator of perceived failures in care quality and professional accountability [2].

Concerns about malpractice may influence clinical decision-making and contribute to defensive medicine, whereby healthcare professionals modify diagnostic or therapeutic practices primarily to reduce perceived legal risk rather than to improve patient care [2,3]. Defensive behaviours may include additional investigations or referrals, more extensive documentation, or avoidance of clinically complex or high-risk situations [3]. In this study, defensive-practice tendencies refer to self-reported or anticipated changes in caution, communication, referral, documentation, treatment selection or clinical avoidance motivated by perceived legal risk. For respondents without independent clinical responsibility, these items represent anticipated responses rather than established clinical behaviour. Defensive medicine is also shaped by organisational culture, institutional policies and wider healthcare-system characteristics [4].

The medico-legal environment also shapes communication, informed consent and clinical documentation, all of which are fundamental components of safe and patient-centred care [5,6]. Informed consent supports shared decision-making and patient autonomy, whilst clear communication and accurate documentation reduce misunderstanding and create an accountable record of clinical reasoning. In dentistry, these issues are especially relevant because care frequently involves procedural interventions, irreversible treatment and expectations concerning function and appearance.

The medico-legal environment also shapes communication, informed consent and clinical documentation, all of which are fundamental components of safe and patient-centred care [5,6]. Rather than representing a purely legal obligation, informed consent supports shared decision-making, promotes trust and helps align clinical care with patients’ values and preferences [5]. Conversely, inadequate communication or incomplete documentation may increase the likelihood of misunderstandings, patient dissatisfaction and malpractice claims. In dental practice, these issues may be particularly relevant because treatment often involves procedural interventions, aesthetic expectations, informed consent for irreversible procedures and close dependence on accurate documentation and patient communication. Consequently, medico-legal preparedness extends beyond knowledge of legislation to include the practical communication and documentation skills required for everyday clinical practice.

Medico-legal education plays a central role in preparing healthcare professionals to navigate increasingly complex clinical and legal environments [7]. Dental students and early-career dentists may encounter medico-legal issues through formal teaching, informal clinical exposure, and observation of everyday professional practice; however, these experiences may be fragmented and insufficient. Although evidence specifically focused on dental students remains limited, studies involving medical trainees suggest that malpractice concerns and defensive behaviours may be perceived as part of the clinical environment even before independent clinical responsibility is assumed [8]. More recent studies involving practising physicians have similarly shown that medico-legal concerns may influence professional attitudes, clinical decision-making and defensive clinical behaviours [9,10]. Collectively, these findings suggest that medico-legal preparedness is likely to evolve throughout dental training and professional practice and is shaped by both formal education and cumulative clinical experience.

Although Romania has a well-established legal framework governing medical malpractice, patients’ rights and informed consent, empirical evidence regarding the medico-legal preparedness of Romanian dental students, dental residents and dentists remains limited [11]. Here, medico-legal preparedness is defined as self-perceived readiness to recognise and respond appropriately to a malpractice-related situation or complaint; it should not be interpreted as objectively tested legal competence. Romanian dental education spans six years, and malpractice-related teaching begins during the early years. Including students from Years I–III was therefore intentional: it allowed awareness and anticipated responses to be examined before extensive clinical responsibility, alongside the perceptions of clinical-year students, residents and practising dentists.

This study aimed to evaluate awareness of malpractice and self-perceived medico-legal preparedness across the full continuum of Romanian dental education and professional practice. It also assessed previous medico-legal education, perceived knowledge, medico-legal concerns and defensive-practice tendencies, and examined whether legal training, professional status, clinical experience, familiarity with malpractice and questionnaire format were associated with these outcomes. The analysis was intended to identify educational needs rather than to equate students’ anticipated responses with dentists’ established clinical behaviour.

## 2. Materials and Methods

### 2.1. Study Design and Setting

A cross-sectional questionnaire-based study was conducted at the Faculty of Dentistry, University of Medicine and Pharmacy of Craiova, Romania, and within its associated educational and professional community. Participants included dental students, dental residents and practising dentists. Data were collected between May and June 2026 using an anonymous, self-administered questionnaire delivered electronically through Google Forms or on paper during a professional conference. This study is reported in accordance with the Strengthening the Reporting of Observational Studies in Epidemiology (STROBE) Statement for cross-sectional studies [12].

### 2.2. Participants and Recruitment

The target population comprised Romanian dental students in Years I–VI, dental residents, specialist dentists and senior-grade dentists (the Romanian professional grade medic dentist primar). Participants were eligible if they were enrolled in dental education or professionally active in dentistry in Romania and voluntarily completed the questionnaire. Residents were treated as a distinct postgraduate training group, whereas specialist and senior-grade dentists were separate source categories that were combined only for inferential analyses because the senior-grade subgroup was small (n = 6).

Convenience sampling was used, meaning that eligible participants were approached through accessible educational and professional networks rather than selected from a probability-based sampling frame. The electronic questionnaire was circulated through the dental academic community, and the paper version was administered to eligible attendees at a professional conference. Because the electronic link was not distributed through a closed invitation list and conference attendance did not provide a defined sampling denominator, a conventional response rate could not be calculated. Participation was voluntary, no financial or material incentives were offered, and submission of the anonymous questionnaire indicated informed consent.

The analytical dataset comprised 176 submitted questionnaires: 134 completed through Google Forms and 42 completed on paper at the conference. Item-level missingness was handled as described below; questionnaires were not removed solely because an individual item was unanswered.

### 2.3. Questionnaire Development

The questionnaire was developed specifically for this study. Items were generated by a member of the research team who teaches malpractice and medico-legal topics to dental students at the Faculty of Dentistry, together with the team’s biostatistician. Content was informed by recurrent issues in dental education and practice, including malpractice, patient communication, informed consent, documentation, professional responsibility and defensive practice.

Before full administration, the questionnaire underwent a preliminary comprehensibility test involving 10 eligible participants. Their feedback led only to minor wording and presentation refinements, affecting no more than approximately 5% of the instrument and not altering item intent, response options or scoring. Because the final meaning and measurement structure remained unchanged, these 10 responses were retained in the analytical dataset.

No formal content-validity-index procedure, independent expert-rating exercise or exploratory factor analysis was performed before or within the present study. Cronbach’s alpha was therefore used only to describe internal consistency and not to establish content validity, construct validity or unidimensionality. The author-developed indices were retained as exploratory composite measures, and their psychometric validation remains a separate objective for future research.

### 2.4. Questionnaire Structure

The questionnaire addressed demographic and professional characteristics, previous legal education, familiarity with malpractice, objective legal-knowledge items, perceived knowledge of the legal framework, perceptions regarding malpractice events and complaint trends, perceived causes and preventive measures, and Likert-scale statements concerning legal awareness, medico-legal fear and defensive-practice tendencies. The complete English version of the questionnaire is provided in Supplementary File S1.

The questionnaire included both single-choice and multiple-choice items. Multiple-choice items allowed respondents to select up to three options, particularly for perceived causes of malpractice and preventive measures considered effective for reducing malpractice risk. Likert-scale items were scored from 1 to 5, with higher values indicating stronger agreement or greater intensity of the construct being assessed, depending on each item’s wording.

The questionnaire was structured into the following domains:

1. Demographic and professional characteristics, including age group, gender, professional status, working environment and clinical experience;
2. Previous exposure to healthcare legislation and sources of medico-legal knowledge;
3. Familiarity with malpractice and perceived knowledge of relevant legal provisions;
4. Perceived preparedness to manage a malpractice-related situation;
5. Perceptions regarding the frequency of malpractice and trends in malpractice complaints;
6. Perceived causes of malpractice and preventive measures;
7. Likert-scale items assessing legal awareness, medico-legal fear and defensive medicine-related behaviours.

### 2.5. Variables and Outcomes

The main outcome was self-perceived preparedness to manage a malpractice-related situation or complaint, measured on an ordinal scale from ‘not prepared at all’ to ‘very well prepared’. This variable represents perceived readiness rather than an objective test of legal knowledge or clinical competence. All response categories were reported descriptively, and the variable was treated as ordinal in inferential analyses.

The main independent variables were previous legal training, professional status, clinical experience, familiarity with malpractice, age group and data source. Previous legal training was analysed both descriptively as a categorical variable and inferentially as a binary variable, distinguishing respondents with any prior legal training from those with none. Legal training included curricular exposure, continuing medical education or both.

Professional status was grouped into four analytically defined categories: early-year students (Years I–III), clinical-year students (Years IV–VI), dental residents and practising dentists. Dental residents were postgraduate trainees and remained separate from independently practising dentists. Specialist dentists (n = 28) and senior-grade dentists (medic dentist primar; n = 6) are distinct Romanian professional grades; they were combined as practising dentists for inferential analysis solely because of the small senior-grade subgroup. The early-year group was included intentionally because malpractice-related content is introduced during Years I and II; and the study aimed to assess awareness before substantial clinical responsibility.

Clinical experience was grouped as no clinical experience, limited clinical experience and professional clinical experience. Limited experience included university clinical placements or less than 1 year of independent practice; professional experience included 1 or more years of practice. The 1-year boundary followed the response categories specified in the questionnaire and was intended to distinguish placements or very early practice from sustained professional exposure, although the professional category remained heterogeneous. Fourteen responses belonged to a legacy coded category that could not be mapped unambiguously during database harmonisation; these were described as undefined and excluded only from analyses requiring grouped clinical experience.

Secondary outcomes included perceived frequency of malpractice events, perceived changes in the number of formal malpractice complaints over the previous 5–10 years, and perceived changes in the frequency with which patients complained. These concepts were kept distinct: malpractice referred to a perceived occurrence involving professional fault and patient harm; a malpractice complaint referred to a formal allegation; and a patient complaint could include dissatisfaction that had not become a formal malpractice claim. Other secondary outcomes were perceived causes, preventive measures and exploratory composite indices of medico-legal fear, defensive-practice tendencies and legal awareness.

### 2.6. Composite Scores

Three exploratory composite indices were calculated from Likert-type items scored from 1 to 5: medico-legal fear (items 25–29), defensive-practice tendencies (items 30–46; 17 items) and legal awareness (items 47, 48, 49, 51, 52 and 54). Each index was the arithmetic mean of its component items when at least 80% of the relevant responses were available. Higher values represented greater intensity of the corresponding construct. No external calibration, diagnostic threshold or categorical interpretation was applied.

The medico-legal fear index captured emotional and cognitive concern about malpractice-related consequences. The defensive-practice index combined reported or anticipated caution, referral, communication, additional investigation or documentation, treatment modification and avoidance motivated by legal risk. The legal-awareness index reflected respondents’ perceived value of legal knowledge, legislation-related confidence and attitudes towards medico-legal education; it was not an objective knowledge test.

Internal consistency was described using Cronbach’s alpha. Alpha was interpreted as evidence of inter-item consistency only and not as proof of validity or a single latent dimension. Because the questionnaire was author-developed and not externally validated, all three indices were treated as exploratory.

### 2.7. Data Management, Cleaning and Handling of Missing Data

Responses collected through Google Forms and paper-based questionnaires were centralised in Microsoft Excel. Excel was used for data entry, database consolidation, coding verification, and uniformisation of the final dataset prior to statistical analysis.

The dataset was checked for duplicate identifiers, missing values, anomalous response patterns and inconsistent coding. No duplicate respondent identifiers were identified. Overall item-level missingness was low and occurred mainly in the paper-based subset. No imputation was performed. Composite indices used the 80% completeness rule, whereas regression models used analysis-specific complete cases.

For descriptive analyses, valid denominators were reported where appropriate. For inferential analyses, respondents with missing values in the variables required for a given test or model were excluded from that specific analysis.

Multiple-choice variables, including perceived causes of malpractice and preventive measures, were converted into binary indicators for each response option. Each option was coded as selected or not selected.

### 2.8. Statistical Analysis

Statistical analyses were performed using IBM SPSS Statistics for Windows, Version 26.0. Microsoft Excel was used for data collection, database consolidation and preliminary data uniformisation.

Descriptive statistics were used to summarise the characteristics of the study population and the distribution of questionnaire responses. Categorical variables were reported as frequencies and percentages. Ordinal and Likert-scale variables were summarised using medians and interquartile ranges, with means and standard deviations also reported to facilitate interpretation. Composite scores were summarised using valid sample size, mean, standard deviation, median and interquartile range.

Bivariate analyses were performed to explore associations between key predictors and outcomes. The Mann–Whitney U test was used to compare ordinal or non-normally distributed continuous variables between two groups. The Kruskal–Wallis test was used for comparisons involving more than two groups, with post-hoc pairwise comparisons performed where appropriate. Spearman’s rank correlation was used to assess associations between ordinal variables and composite scores. Chi-square or Fisher’s exact tests were used for associations between categorical variables, depending on expected cell counts.

Effect sizes were reported where appropriate. Rank-biserial correlation was used for Mann–Whitney U tests, epsilon squared for Kruskal–Wallis tests, Cramer’s V for categorical associations and Spearman’s rho for rank correlations. To account for multiple exploratory comparisons, *p*-values were adjusted using the Benjamini–Hochberg false-discovery-rate procedure across the 15 bivariate comparisons performed.

A multivariable ordinal logistic regression model was used to identify factors independently associated with perceived preparedness to manage malpractice-related situations. The model included professional status, clinical experience, previous legal training, familiarity with malpractice, age group and data source as predictors. Adjusted odds ratios, 95% confidence intervals and *p*-values were reported.

Two robust linear regression models were used to explore factors associated with the defensive-practice and legal-awareness indices. Coefficients were estimated by ordinary least squares with heteroscedasticity-consistent (HC3) robust standard errors. The defensive-practice model included previous legal training, familiarity with malpractice, perceived frequency of malpractice, perceived complaint trend and data source and was fitted on 170 respondents. The legal-awareness model included professional clinical experience, previous legal training, familiarity with malpractice, age group and data source and was fitted on 172 respondents. Model fit was assessed using R2 and adjusted R2.

As requested during peer review, a sensitivity analysis excluded early-year students and retained clinical-year students, residents and practising dentists (n = 68). To reduce overfitting and avoid modelling professional status and clinical experience simultaneously in the smaller subgroup, the ordinal preparedness model included professional group, previous legal training, familiarity with malpractice and data source. The defensive-practice model retained the predictors used in the main robust regression. These analyses evaluated whether the principal associations persisted among respondents with clinical exposure.

All statistical tests were two-tailed, and statistical significance was set at *p* < 0.05.

### 2.9. Ethical Considerations

The study was conducted in accordance with the principles of the Declaration of Helsinki. Participation was voluntary and anonymous, and data were analysed only in aggregated form. No personally identifiable information was collected in the analytical dataset.

Ethical approval was obtained from the Ethics Committee of the University of Medicine and Pharmacy of Craiova under approval number 76, dated 21 April 2026.

## 3. Results

### 3.1. Study Population and Data Completeness

A total of 176 responses were included in the analysis. Of these, 134 (76.1%) were collected through Google Forms and 42 (23.9%) on paper at a professional conference. The two formats reached different participant profiles: 36 of 42 paper respondents were residents or practising dentists, whereas 118 of 134 online respondents were students. Overall item-level missingness was low and occurred predominantly in the paper-based subset.

The sample was predominantly young and student-based. Ninety-four respondents (53.4%) were aged 18–23 years, followed by 32 (18.2%) aged 31–40 years, 24 (13.6%) aged 24–30 years, 20 (11.4%) aged 41–50 years and six (3.4%) aged over 50 years. Among valid gender responses, 126 participants were female (72.4%), 44 were male (25.3%), and four preferred not to disclose their gender (2.3%).

Regarding professional status, 124 respondents (70.5%) were students and 52 (29.5%) were dental residents or practising dentists. After grouping by training stage, 108 participants (61.4%) were early-year students (Years I–III), 16 (9.1%) were clinical-year students (Years IV–VI), 18 (10.2%) were dental residents, and 34 (19.3%) were specialist or senior-grade dentists. More than half reported no clinical activity (n = 92; 52.3%), 40 (22.7%) had limited exposure, and 30 (17.0%) had professional clinical experience. Fourteen (8.0%) had an undefined legacy experience code and were excluded from models requiring grouped clinical experience.

The demographic and professional characteristics of the respondents are summarised in Table 1.

### 3.2. Legal Education, Familiarity with Malpractice and Perceived Preparedness

Formal exposure to healthcare legislation was limited. Overall, 110 participants (62.5%) reported no previous legal training, whereas 44 (25.0%) reported training within the curriculum, 16 (9.1%) through continuing medical education and six (3.4%) through both routes. Although 108 respondents (61.4%) stated they were familiar with the concept of malpractice, 54 (30.7%) had only heard of it without knowing the details, and 14 (8.0%) reported no familiarity.

Knowledge of the legal framework appeared heterogeneous. The most frequently selected response regarding malpractice regulation was ‘I do not know’ (n = 82; 46.6%), followed by Law no. 95/2006 (n = 68; 38.6%). Regarding perceived preparedness to manage a malpractice-related situation, only 14 respondents (8.0% of valid responses) considered themselves well or very well prepared. Most respondents reported low or moderate preparedness: 50 (28.7% of valid responses) reported not being prepared at all, 58 (33.3%) reported being slightly prepared, and 52 (29.9%) reported being moderately prepared.

Legal education, familiarity with malpractice and perceived preparedness are summarised in Table 2.

### 3.3. Perceptions Regarding the Frequency and Evolution of Malpractice

Most respondents perceived malpractice as a relevant phenomenon. Fourteen respondents (8.0%) considered malpractice very frequent, and 36 (20.5%) frequent, while the most common response was ‘moderate’ (n = 72; 40.9%). A further 20 participants (11.4%) considered malpractice to be rare, and 34 (19.3%) answered ‘I do not know’.

Perceptions regarding complaint trends suggested an increasing medico-legal pressure. Ninety-six respondents (54.5%) considered that malpractice complaints had increased either significantly (n = 32; 18.2%) or slightly (n = 64; 36.4%) over the previous 5–10 years. Similarly, 94 respondents (53.4%) believed that patients were complaining more frequently, either significantly (n = 30; 17.0%) or slightly (n = 64; 36.4%).

### 3.4. Perceived Causes of Malpractice and Preventive Measures

The most frequently selected perceived causes of malpractice were insufficient training (n = 102; 58.0%), negligence (n = 96; 54.5%), lack of experience (n = 82; 46.6%), deficient communication (n = 82; 46.6%) and absence of informed consent (n = 72; 40.9%). Less frequently selected causes included insufficient knowledge of legislation (n = 44; 25.0%), unrealistic expectations (n = 32; 18.2%), overload (n = 28; 15.9%) and insufficient technical resources (n = 26; 14.8%).

The preventive measures most often considered effective were documentation (n = 92; 52.3%), continuing education (n = 90; 51.1%), protocols (n = 88; 50.0%), communication (n = 84; 47.7%) and mandatory courses (n = 82; 46.6%). Professional supervision and malpractice insurance were selected by 50 (28.4%) and 52 (29.5%) respondents, respectively, while legal-framework reform was selected by 22 respondents (12.5%).

The perceived causes of malpractice and preventive measures selected by respondents are presented in Table 3.

### 3.5. Attitudes, Medico-Legal Fear and Defensive Medicine-Related Behaviours

Likert-scale responses indicated the strongest agreement with statements supporting the importance of legal knowledge. The item ‘knowledge of legislation reduces malpractice risk’ had the highest mean score (mean = 3.66, SD = 1.12; median = 4, IQR 3.00–5.00), followed by the view that students should study legislation (mean = 3.53, SD = 0.94; median = 4, IQR 3.00–4.00) and that legal knowledge increases professional confidence (mean = 3.36, SD = 1.04; median = 3, IQR 3.00–4.00).

By contrast, lower scores were observed for behaviours involving omission or avoidance, including omitting information about minor complications (mean = 2.02, SD = 1.13; median = 2, IQR 1.00–3.00), choosing a simpler treatment plan (mean = 2.12, SD = 1.08; median = 2, IQR 1.00–3.00) and avoiding the expression of uncertainty (mean = 2.20, SD = 1.11; median = 2, IQR 1.00–3.00). The highest defensive-behaviour item was increased caution in clinical decisions (mean = 3.26, SD = 1.06; median = 3, IQR 3.00–4.00).

The exploratory indices showed good internal consistency. Cronbach’s alpha was 0.842 for medico-legal fear, 0.919 for defensive-practice tendencies and 0.834 for legal awareness. The recalculated 17-item defensive-practice index had a mean of 2.60 (SD = 0.74; median = 2.59, IQR 2.06–3.06), whilst the legal-awareness index had a mean of 3.29 (SD = 0.70; median = 3.33, IQR 3.00–3.83). Internal consistency does not, however, establish the validity or unidimensionality of these author-developed indices.

The internal consistency and descriptive statistics for the composite scores are shown in Table 4.

### 3.6. Bivariate Analyses

Previous legal training was significantly associated with greater perceived preparedness to manage a malpractice situation. Respondents with legal training had a median preparedness score of 3.00 (IQR 2.00–3.00), compared with 2.00 (IQR 1.00–2.00) among those without legal training (Mann–Whitney U = 1990.0, rank-biserial r = 0.442, *p* < 0.001; Benjamini–Hochberg-adjusted *p* < 0.001). Familiarity with malpractice was also positively associated with preparedness (Spearman’s rho = 0.230, *p* = 0.002; adjusted *p* = 0.010).

Perceived preparedness differed significantly by professional status (Kruskal–Wallis H = 51.53, epsilon squared = 0.285, *p* < 0.001; adjusted *p* < 0.001). Median preparedness was 2.00 (IQR 1.00–2.00) in preclinical students, 2.50 (IQR 1.75–3.00) in clinical students, 3.00 (IQR 2.00–3.00) in residents and 3.00 (IQR 3.00–3.00) in specialists or senior dentists. Post hoc comparisons showed significantly lower preparedness among preclinical students compared with specialists/senior dentists (adjusted *p* < 0.001) and residents (adjusted *p* < 0.001). Specialists/senior dentists also had higher preparedness than clinical students (adjusted *p* = 0.013).

Clinical experience was not significantly associated with the defensive medicine score in the bivariate analysis (Kruskal–Wallis H = 0.63, *p* = 0.731; adjusted *p* = 0.832), with a negligible effect size. Similarly, perceived increases in complaints over the previous 5–10 years and perceived frequency of patient complaints were not significantly correlated with the defensive medicine score (both adjusted *p* > 0.89). Legal training was not significantly associated with the legal awareness score (*p* = 0.219; adjusted *p* = 0.381), and familiarity with malpractice showed no significant bivariate association with legal awareness (rho = 0.075, *p* = 0.326; adjusted *p* = 0.513).

Exploratory comparisons of legal-information sources by student versus dentist/dental resident status showed significant differences for continuing medical education (Cramer’s V = 0.397, adjusted *p* < 0.001), literature (Cramer’s V = 0.378, adjusted *p* < 0.001), mass media (Cramer’s V = 0.259, adjusted *p* = 0.003) and having no sources of legal knowledge (Cramer’s V = 0.229, adjusted *p* = 0.010). Perceived causes of malpractice were broadly similar between students and dentists/dental residents, with only insufficient knowledge of legislation remaining significant after adjustment (Cramer’s V = 0.187, adjusted *p* = 0.043). In the analysis of preventive measures by clinical experience, mandatory courses (Cramer’s V = 0.250, adjusted *p* = 0.023) and supervision (Cramer’s V = 0.310, adjusted *p* = 0.003) remained significant after adjustment.

The main bivariate analyses are summarised in Table 5.

### 3.7. Multivariable Models

In the multivariable ordinal logistic regression model for perceived preparedness (complete-case n = 160), previous legal training remained independently associated with higher preparedness after adjustment for professional status, clinical experience, familiarity with malpractice, age and data source (adjusted OR = 2.52, 95% CI 1.03–6.15, *p* = 0.042). Residents had higher odds of reporting greater preparedness compared with preclinical students (adjusted OR = 8.15, 95% CI 1.08–61.72, *p* = 0.042), and respondents with professional clinical experience had higher odds compared with those with no clinical experience (adjusted OR = 6.11, 95% CI 1.11–33.77, *p* = 0.038). Limited clinical experience showed a borderline association (adjusted OR = 2.13, 95% CI 0.95–4.78, *p* = 0.065).

The multivariable model for the 17-item defensive-practice index explained a limited proportion of variance (R2 = 0.066; adjusted R2 = 0.037; robust Wald *p* = 0.024). After adjustment, only data source was significantly associated with the index: paper respondents reported higher scores than online respondents (β = 0.47, 95% CI 0.19 to 0.75, *p* = 0.001). Previous legal training, familiarity with malpractice, perceived frequency of malpractice and perceived complaint trend were not significant in the full cohort. Because paper administration occurred at a conference and predominantly reached residents and practising dentists, this association may reflect respondent composition, administration context, residual confounding or a combination of these factors; it should not be interpreted as an effect of questionnaire format alone.

For the legal awareness score, the overall model was statistically significant but explained a modest proportion of variance (R^2^ = 0.077; adjusted R^2^ = 0.049; model *p* < 0.001). Greater familiarity with malpractice was independently associated with a higher legal awareness score (β = 0.18, 95% CI 0.03 to 0.32, *p* = 0.020), and respondents in the paper-based subset had higher scores than those completing the online questionnaire (β = 0.45, 95% CI 0.19 to 0.70, *p* = 0.001). Professional clinical experience (β = 0.26, *p* = 0.059), previous legal training (β = −0.20, *p* = 0.053) and age group (β = −0.12, *p* = 0.075) showed associations that did not reach conventional statistical significance.

In the sensitivity analysis excluding early-year students (n = 68), practising dentists had higher odds of greater preparedness than clinical-year students (adjusted OR = 5.63, 95% CI 1.53–20.67, *p* = 0.009). Resident status (adjusted OR = 3.97, *p* = 0.162), previous legal training (adjusted OR = 2.02, *p* = 0.247), familiarity with malpractice (adjusted OR = 1.46, *p* = 0.439) and paper participation (adjusted OR = 1.49, *p* = 0.517) were not significant. In the corresponding defensive-practice model (n = 66), familiarity with malpractice (β = 0.50, 95% CI 0.21 to 0.79, *p* < 0.001) and paper participation (β = 0.65, 95% CI 0.23 to 1.07, *p* = 0.002) were associated with higher scores; previous legal training showed a non-significant inverse association (β = −0.39, *p* = 0.063).

The ordinal logistic regression model for perceived preparedness is presented in Table 6, and the robust linear regression models are summarised in Table 7.

### 3.8. Summary of Main Findings

Overall, the findings indicate a gap between recognition of the importance of healthcare legislation and self-perceived preparedness to manage malpractice-related situations. In the main analysis, preparedness was higher among residents, respondents with professional clinical experience and those with previous legal training. The author-developed indices showed good internal consistency but remain exploratory and unvalidated. Sensitivity analysis indicated that some associations differed after early-year students were excluded, reinforcing the need to distinguish awareness and anticipated responses from established clinical behaviour.

## 4. Discussion

### 4.1. Principal Findings

This cross-sectional study assessed malpractice-related awareness, self-perceived medico-legal preparedness, medico-legal fear and defensive-practice tendencies across Romanian dental education and practice. The principal finding was a discrepancy between moderate legal awareness and limited self-perceived readiness to manage malpractice-related situations. The inclusion of early-year students was intentional because the study sought to examine awareness across the educational continuum, including stages at which malpractice-related teaching has begun but substantial clinical responsibility has not yet been assumed.

Perceived preparedness was not evenly distributed across the study population. In bivariate analyses, preparedness was significantly associated with previous legal training, familiarity with malpractice and professional status. In the multivariable ordinal logistic regression model, previous legal training, residency status and professional clinical experience remained independently associated with higher perceived preparedness. These findings suggest that medico-legal preparedness is likely to be shaped by both educational exposure and progressive clinical responsibility.

The exploratory indices showed good internal consistency, but this does not establish content validity, construct validity or unidimensionality. The multivariable models explained only modest variance, suggesting that institutional culture, speciality-specific risk, previous exposure to complaints, communication climate, local legal expectations and tolerance for uncertainty may also influence responses. The indices should therefore be interpreted as descriptive, author-developed measures rather than validated psychometric scales.

### 4.2. The Gap Between Legal Awareness and Perceived Preparedness

One of the most relevant findings of this study is the gap between respondents’ recognition of the importance of healthcare legislation and their limited perceived preparedness to manage malpractice-related situations. The highest Likert-scale scores were observed for statements indicating that knowledge of legislation may reduce malpractice risk, that students should study healthcare legislation and that legal knowledge may increase professional confidence. Nevertheless, only a minority of respondents reported feeling well or very well prepared to manage a malpractice-related situation.

This discrepancy is consistent with recent literature suggesting that students in health-related fields may recognise the importance of medico-legal competence yet feel insufficiently prepared to address legal issues in clinical practice. A recent systematic review of medical law and medical school curricula concluded that medical law is inconsistently represented in medical education and that students may benefit from more structured exposure to legal concepts relevant to clinical care [7]. Similarly, a pilot study evaluating students’ knowledge of medical malpractice highlighted the need for improved educational strategies addressing malpractice-related knowledge during health-professions training [13]. These findings are consistent with the present study, in which familiarity with the concept of malpractice did not necessarily translate into high perceived preparedness.

The Romanian context is particularly relevant. A previous Romanian cross-sectional study reported insufficient physician knowledge of legal provisions governing informed consent and confidentiality, both of which are central elements of the clinician–patient relationship [11]. Our findings extend this evidence by showing that limited medico-legal preparedness is also apparent among dental students, dental residents and practising dentists. Although the professional context of dentistry has its own procedural, communicational and aesthetic dimensions, the medico-legal challenges identified in broader healthcare literature remain highly relevant to dental practice.

Awareness alone may not be sufficient to support effective medico-legal decision-making. Dental professionals and trainees may understand that legal knowledge is important, yet still lack the practical skills to apply it in real clinical contexts. These skills include obtaining and documenting informed consent, communicating uncertainty, managing adverse events, maintaining appropriate clinical records, understanding professional liability and knowing the institutional pathways to follow when a complaint occurs. In dentistry, this distinction is particularly important because medico-legal preparedness also involves understanding the legal implications of consent, professional liability, patient explanations and clinical documentation [14,15,16,17]. Therefore, the gap identified in this study should be viewed not only as a knowledge deficit, but also as a practical preparedness deficit.

### 4.3. Legal Training as a Modifiable Educational Factor

In the main analysis, previous legal training was independently associated with higher self-perceived preparedness after adjustment for professional status, clinical experience, familiarity with malpractice, age and data source. This association did not remain statistically significant in the smaller sensitivity subgroup restricted to participants with clinical exposure. The full-cohort result should therefore be interpreted as evidence of an association across the educational continuum, not as proof that legal training independently predicts preparedness among clinically active respondents.

The association between previous legal training and preparedness does not establish causality, given the cross-sectional design. However, it supports the hypothesis that exposure to healthcare legislation may contribute to greater confidence in managing medico-legal issues. This interpretation is supported by educational literature arguing for the formal integration of medical law into healthcare education, emphasising that legal knowledge is increasingly necessary for safe and ethically sound clinical practice [18]. Moreover, situation-based and interdisciplinary medical ethics and law curricula have been shown to support students’ motivation to learn, professionalism, and understanding of legal and ethical responsibilities [19].

The implication for Romanian dental education is that medico-legal training should not be limited to theoretical knowledge of legislation. The present results support the need for applied educational strategies, including case-based learning, simulated malpractice scenarios, documentation exercises, informed-consent workshops, communication training and interdisciplinary teaching involving dental clinicians, legal professionals and ethicists. Such approaches may be more useful than traditional lecture-based teaching alone, particularly for preparing students and young dentists to manage complex real-life situations. This is consistent with dental medico-legal literature emphasising that dentists require not only knowledge of legal rules but also practical competence in consent, record keeping, explanation of treatment risks and risk-management procedures [14,15,16,17]. In dental practice, where procedures are often invasive, expectations may be high, and aesthetic outcomes are highly visible, applied medico-legal education may have particular value.

### 4.4. Professional Status, Clinical Experience and Medico-Legal Preparedness

Professional status and clinical experience were associated with perceived preparedness in the main cohort. Residents and respondents with professional clinical experience reported greater preparedness than the corresponding reference groups. In the sensitivity analysis excluding early-year students, practising dentists had higher odds of greater preparedness than clinical-year students (adjusted OR = 5.63, 95% CI 1.53–20.67, *p* = 0.009), whereas resident status and previous legal training were not independently significant. The wide confidence intervals indicate limited precision and require cautious interpretation.

Lower preparedness amongst early-year students was expected to some extent because their clinical responsibility was limited. Their inclusion nevertheless addressed a distinct educational question: whether students recognise malpractice-related issues before extensive patient care. Malpractice-related content is introduced during Years I and II, and several defensive-practice items were deliberately phrased as behaviours respondents had performed or would consider performing. Consequently, early-year responses represent awareness and anticipated tendencies, not established clinical practice.

These findings suggest that medico-legal education should be longitudinal. Introductory concepts could be taught early in dental school, while more applied content should be integrated during clinical years, residency and continuing professional development. Early exposure may increase awareness, whereas later training can focus on practical decision-making, documentation, informed consent, complaint management and risk prevention. This staged approach is consistent with the wider literature supporting progressive, context-based medico-legal education rather than isolated or purely theoretical teaching [7,18,19].

### 4.5. Defensive Medicine-Related Behaviour

The 17-item defensive-practice index showed very good internal consistency, although it remains an exploratory composite measure. In the full model, paper participation was associated with a higher score, whilst legal training, familiarity, perceived malpractice frequency and complaint trend were not significant. In the clinically exposed subgroup, familiarity with malpractice (β = 0.50, *p* < 0.001) and paper participation (β = 0.65, *p* = 0.002) were associated with stronger defensive-practice tendencies. Because the paper questionnaire was administered at a conference and reached a more professionally experienced cohort, source-related effects cannot be separated fully from respondent composition and administration context.

Defensive medicine is a complex and multidimensional phenomenon. In the European literature, defensive medicine has been defined in various ways and may include both assurance behaviours, such as ordering additional investigations or documenting more extensively, and avoidance behaviours, such as avoiding high-risk patients or procedures [20]. An international review of empirical research similarly described defensive medicine as practices performed to reduce perceived legal risk, such as additional testing, referrals, and other forms of “hedging” behaviour [3]. More recently, a systematic review and meta-analysis reported a high global prevalence of defensive medicine among physicians and identified multiple determinants, including medico-legal concerns, communication factors, and healthcare system characteristics [21]. Evidence specific to dentistry also supports the relevance of this construct. A recent study on dentists’ malpractice fears and defensive dentistry attitudes reported a close relationship between fear of malpractice and defensive dental behaviour, supporting the need to examine defensive medicine-related responses within dental practice specifically [22].

The limited explanatory power of our defensive medicine model is therefore plausible. Recent qualitative and systematic evidence indicates that defensive practice is shaped by broader professional, organisational, social and cultural factors, not only by fear of litigation [23,24]. Eftekhari et al. identified organisational-managerial, social, and personal factors contributing to defensive medicine [24], whilst Lorenc et al. emphasised that defensive practice may reflect wider concerns about clinical work, institutional support, and professional vulnerability [23]. In addition, a German cross-sectional survey among general practitioners found that fears of perceived legal consequences influenced defensive medical practice, but such behaviours were embedded in a wider context of uncertainty and clinical decision-making [25].

In this context, the present findings should not be interpreted as evidence that defensive medicine is absent or unimportant in the Romanian dental sample. Rather, they suggest that the predictors included in the present questionnaire captured only part of a more complex phenomenon. Defensive medicine in dentistry may be influenced by additional factors not fully captured in this study, such as the type of dental speciality, perceived patient expectations, prior exposure to complaints, fear of reputational damage, availability of institutional support, workload, communication difficulties, and trust in complaint-resolution mechanisms. Future studies should assess these dimensions more directly. The professional and personal impact of formal complaints on dentists should also be considered, as complaint-related experiences may affect clinical confidence, professional behaviour and perceived vulnerability in practice [26].

### 4.6. Perceived Causes of Malpractice and Preventive Measures

Respondents most frequently identified insufficient training, negligence, lack of experience, deficient communication and absence of informed consent as perceived causes of malpractice. These findings are relevant because they link malpractice prevention not only to individual legal knowledge, but also to broader dimensions of clinical competence, communication and patient safety. This is particularly important in dentistry, where clinical procedures often require patient cooperation, detailed explanations, realistic expectation management and accurate documentation of treatment options, risks and outcomes.

The prominence of communication and informed consent in the respondents’ answers is consistent with European and international literature. A large Dutch analysis of medical liability claims reported that ineffective clinician–patient communication, lack of informed consent, and unrealistic treatment expectations may increase the risk of malpractice claims [27]. Informed consent has also increasingly been conceptualised not merely as a legal requirement but as a cornerstone of the care relationship and patient autonomy [5]. A comparative European study on informed consent regulation emphasised the importance of clear communication about treatment, alternatives, and major risks, preferably supported by written documentation [6]. Dental-specific studies similarly indicate that inadequate patient information, insufficient explanation of risks and failures in informed consent may contribute to malpractice disputes [15,17,28].

The preventive measures most frequently selected in the present study were documentation, continuing education, protocols, communication and mandatory courses. This pattern suggests that respondents recognise the importance of both individual and system-level strategies. Documentation and communication are closely linked to daily dental practice, whereas protocols and continuing education reflect institutional and professional responsibilities. The importance of documentation is also supported by the dental literature, which describes dental records as both a professional obligation and a key element of medico-legal protection for dentists and patients [16]. Mandatory courses in healthcare legislation were also frequently selected, reinforcing the central message that medico-legal education is perceived as necessary.

These findings have practical implications. Malpractice prevention should not be framed exclusively as a legal defence strategy. Instead, it should be integrated into patient-safety education, communication training, clinical governance and quality-improvement programmes. This is consistent with the broader literature on malpractice liability and healthcare quality, which suggests that liability pressure alone is unlikely to be a reliable mechanism for improving care quality and that more direct quality-improvement strategies are needed [29]. In dental education and dental practice, this means that medico-legal preparedness should be integrated into everyday clinical routines, including treatment planning, informed consent, record-keeping, patient communication, and the management of dissatisfaction or adverse outcomes.

### 4.7. Implications for Romanian Dental Education and Dental Practice

The large proportion of early-year students limits generalisability to Romanian practising dentists but is informative for the study’s educational objective. These participants had already encountered malpractice-related teaching in Years I and II, allowing awareness and anticipated responses to be evaluated before extensive clinical responsibility. The sensitivity analysis restricted to clinical-year students, residents and practising dentists was added to determine which findings persisted in a clinically exposed subgroup.

A staged educational model is therefore appropriate. During the early years, existing malpractice teaching can establish concepts of professional responsibility, patient rights, informed consent and professional duties. During clinical years, the emphasis can move towards documentation, communication, adverse-event management and applied case discussion. Residency and continuing professional development can address speciality-specific risks, complaint management, institutional procedures, expert evaluation and interdisciplinary collaboration with legal professionals.

Such an approach would be consistent with the broader need to prepare future dentists not only for biomedical decision-making but also for the ethical, legal, and communicative responsibilities of dental practice. In this sense, medico-legal education should be considered part of professional competence, rather than an optional or peripheral component of dental training. The Romanian evidence on gaps in legal knowledge regarding informed consent and confidentiality [11], together with the present findings on limited preparedness, supports the need for curriculum-level and continuing-education interventions. Importantly, such interventions should be practical and clinically oriented, rather than limited to the passive transmission of legislative information. Recent dental literature on medico-legal risk and orthodontic malpractice further supports the need for speciality-sensitive risk-management training, particularly in areas where patient expectations, treatment duration, aesthetic outcomes and communication demands may increase vulnerability to disputes [30].

### 4.8. Strengths and Limitations

This study addresses an underexplored topic in Romania, includes participants from successive educational and professional stages, and combines descriptive, bivariate, multivariable and sensitivity analyses. The inclusion of early-year students permits awareness to be examined before extensive clinical responsibility, whilst the clinically exposed subgroup provides a more practice-oriented comparison. The transparent description of the author-developed indices and their limitations is also important for interpreting the results.

Several limitations should be acknowledged. First, convenience sampling and recruitment from a single academic-professional setting preclude national representativeness. Second, early-year students constituted 61.4% of the sample. Their responses are relevant to awareness and anticipated tendencies but cannot be interpreted as evidence of established clinical behaviour. Third, the cross-sectional design does not support causal inference, and associations between legal training and preparedness remain exploratory.

Fourth, the data were self-reported and may be affected by recall, social-desirability and interpretation biases. Fifth, the questionnaire underwent only a preliminary comprehensibility test with 10 participants, whose responses were retained after minor non-substantive refinements. No formal content-validity-index assessment, exploratory factor analysis, independent external validation or test–retest assessment was performed. Cronbach’s alpha therefore describes internal consistency only. Sixth, professional status and clinical experience overlap conceptually, the ≥1-year experience category is heterogeneous, and the corresponding multivariable estimates were imprecise. Seventh, online and conference-based paper administration reached different participant profiles; adjustment for data source cannot exclude residual confounding or mode and context effects. Finally, perceptions of complaint trends from respondents without professional experience may be speculative.

### 4.9. Future Research

Future research should include larger, multicentre samples with a more balanced distribution of dental students, dental residents and dentists across specialities and regions. Longitudinal studies would be useful for assessing whether medico-legal education improves preparedness, communication behaviour, documentation quality, and confidence in managing malpractice-related situations. Future studies should also explore speciality-specific differences, prior exposure to complaints, institutional support, the perceived legal climate, and the relationship between medico-legal fear and actual clinical decision-making.

Further validation should include independent expert review, formal content-validity assessment, cognitive interviewing, exploratory and confirmatory factor analysis in separate samples, and test–retest reliability. Larger multicentre studies should use more balanced educational and professional groups and distinguish anticipated responses from documented clinical behaviour. Qualitative work could also clarify how students and practitioners understand malpractice events, formal malpractice complaints and broader patient dissatisfaction.

### 4.10. Overall Interpretation

Overall, Romanian dental students, residents and practising dentists recognised the relevance of healthcare legislation but reported limited preparedness to manage malpractice-related situations. In the main cohort, previous legal training, resident status and professional clinical experience were associated with preparedness. In the clinically exposed subgroup, practising-dentist status remained associated with greater preparedness, whereas familiarity with malpractice was associated with stronger defensive-practice tendencies. These subgroup findings temper, rather than invalidate, the full-cohort interpretation.

The study therefore contributes to the limited evidence on medico-legal preparedness in Romanian dental education and practice. It highlights that legal awareness, although important, is not sufficient on its own. Dental trainees and practitioners also need practical skills, institutional guidance and repeated opportunities to apply medico-legal principles in clinically realistic contexts. In this way, medico-legal education may support not only risk reduction, but also better communication, safer clinical practice and more confident professional decision-making.

## 5. Conclusions

This cross-sectional study identified a substantial gap between the perceived importance of healthcare legislation and self-reported preparedness to manage malpractice-related situations among Romanian dental students, dental residents and dentists. Although respondents generally recognised the relevance of legal knowledge in reducing malpractice risk and increasing professional confidence, most had not received previous formal legal training, and only a small proportion considered themselves well or very well prepared to manage a malpractice-related situation.

In the main analysis, previous legal training, resident status and professional clinical experience were independently associated with higher self-perceived preparedness. Sensitivity analysis showed that, among clinically exposed participants, practising dentists reported greater preparedness than clinical-year students, while familiarity with malpractice and conference-based paper participation were associated with stronger defensive-practice tendencies. These findings indicate that educational stage, professional exposure and data-collection context must all be considered when interpreting the results.

The results support the integration of structured, applied and longitudinal medico-legal education into Romanian dental curricula, residency training and continuing professional development programmes. Such training should include not only theoretical legal concepts but also practical components related to informed consent, documentation, patient communication, adverse event management, and institutional risk prevention. Larger multicentre studies and longitudinal educational interventions are needed to validate these findings and to assess whether structured medico-legal training can improve preparedness, confidence and clinical risk-management behaviours.

## Figures and Tables

**Table 1:** Demographic and professional characteristics of the respondents.

Characteristic	n	%
Data source		
Google Forms	134	76.1
Paper-based questionnaire	42	23.9
Age group		
18–23 years	94	53.4
24–30 years	24	13.6
31–40 years	32	18.2
41–50 years	20	11.4
>50 years	6	3.4
Gender		
Female	126	72.4 *
Male	44	25.3 *
Prefer not to answer	4	2.3 *
Missing	2	1.1
Professional group		
Early-year student (Years I–III)	108	61.4
Clinical-year student (Years IV–VI)	16	9.1
Resident	18	10.2
Specialist or senior-grade dentist	34	19.3
Clinical experience		
No clinical experience	92	52.3
Limited clinical experience	40	22.7
Professional clinical experience	30	17.0
Undefined legacy category	14	8.0

* Percentages for gender categories are calculated among valid responses, except for missing values.

**Table 2:** Legal education, familiarity with malpractice and perceived preparedness.

Variable/Category	n	%
Legal training		
No previous legal training	110	62.5
Curricular training	44	25.0
Continuing medical education	16	9.1
Both curricular and CME	6	3.4
Familiarity with malpractice		
Familiar	108	61.4
Heard of it, but without details	54	30.7
Not familiar	14	8.0
Perceived preparedness		
Not prepared at all	50	28.7 *
Slightly prepared	58	33.3 *
Moderately prepared	52	29.9 *
Well prepared	12	6.9 *
Very well prepared	2	1.1 *

* Percentages for perceived preparedness are calculated among valid responses (n = 174). CME: continuing medical education.

**Table 3:** Perceived causes of malpractice and preventive measures.

Type	Perceived Cause or Preventive Measure	n	%
Cause	Insufficient training	102	58.0
	Negligence	96	54.5
	Lack of experience	82	46.6
	Deficient communication	82	46.6
	Absence of informed consent	72	40.9
	Insufficient knowledge of legislation	44	25.0
	Unrealistic expectations	32	18.2
	Overload	28	15.9
	Technical resources	26	14.8
Preventive measure	Documentation	92	52.3
	Continuing education	90	51.1
	Protocols	88	50.0
	Communication	84	47.7
	Mandatory courses	82	46.6
	Malpractice insurance	52	29.5
	Professional supervision	50	28.4
	Legal-framework reform	22	12.5

Percentages are calculated from the total sample (n = 176). Respondents could select up to three options.

**Table 4:** Internal consistency and descriptive statistics for composite scores.

Score	Valid n	Cronbach Alpha	Mean	SD	Median	IQR
Medico-legal fear score	172	0.84	2.68	0.87	2.60	2.00–3.20
Defensive-practice tendency score	170	0.92	2.60	0.74	2.59	2.06–3.06
Legal awareness score	172	0.83	3.29	0.70	3.33	3.00–3.83

Indices were calculated as the mean of their component items when at least 80% of the relevant responses were available. Cronbach’s alpha was calculated among respondents with complete data for the corresponding item set.

**Table 5:** Main bivariate analyses.

Predictor	Outcome	Test	Statistic	Effect Size	*p*-Value	Adjusted *p*-Value
Legal training	Perceived preparedness	Mann–Whitney U	1990.0	r = 0.442	<0.001	<0.001
Familiarity with malpractice	Perceived preparedness	Spearman	rho = 0.230	rho = 0.230	0.002	0.010
Professional status	Perceived preparedness	Kruskal–Wallis	H = 51.53	ε^2^ = 0.285	<0.001	<0.001
Clinical experience	Defensive medicine score	Kruskal–Wallis	H = 0.63	ε^2^ ≈ 0.000	0.731	0.832
Complaint trend	Defensive medicine score	Spearman	rho = −0.007	rho = −0.007	0.924	0.968
Patient complaint frequency	Defensive medicine score	Spearman	rho = 0.018	rho = 0.018	0.813	0.894
Legal training	Legal awareness score	Mann–Whitney U	3794.0	r = 0.113	0.219	0.381
Familiarity with malpractice	Legal awareness score	Spearman	rho = 0.075	rho = 0.075	0.326	0.513

Adjusted *p*-values were obtained using the Benjamini–Hochberg false-discovery-rate procedure. Rank-biserial correlation was reported in the direction of the observed association, indicating higher preparedness among respondents with previous legal training.

**Table 6:** Multivariable ordinal logistic regression model for perceived preparedness to manage malpractice situations.

Predictor	Adjusted OR	95% CI	*p*-Value
Specialist/senior-grade dentist vs. early-year student	1.58	0.22–11.12	0.647
Clinical-year student vs. early-year student	1.00	0.28–3.55	0.997
Resident vs. early-year student	8.15	1.08–61.72	0.042
Limited clinical experience vs. none	2.13	0.95–4.78	0.065
Professional clinical experience vs. none	6.11	1.11–33.77	0.038
Previous legal training vs. none	2.52	1.03–6.15	0.042
Familiarity with malpractice	1.37	0.83–2.27	0.218
Age group	1.06	0.72–1.55	0.783
Paper-based questionnaire vs. Google Forms	0.30	0.08–1.07	0.063

Reference categories: preclinical student, no clinical experience, no previous legal training and Google Forms data source. OR: odds ratio; CI: confidence interval.

**Table 7:** Adjusted coefficients from robust linear regression models for defensive medicine and legal awareness scores.

Outcome	Predictor	β	95% CI	*p*-Value
Defensive-practice tendency score	Previous legal training vs. none	−0.16	−0.42 to 0.10	0.219
	Familiarity with malpractice	0.11	−0.08 to 0.30	0.266
	Perceived frequency of malpractice	−0.01	−0.12 to 0.11	0.928
	Perceived complaint trend	−0.01	−0.10 to 0.07	0.748
	Paper-based questionnaire vs. Google Forms	0.47	0.19 to 0.75	0.001
Legal awareness score	Professional clinical experience vs. no professional experience	0.26	−0.01 to 0.52	0.059
	Previous legal training vs. none	−0.20	−0.40 to 0.00	0.053
	Familiarity with malpractice	0.18	0.03 to 0.32	0.020
	Age group	−0.12	−0.24 to 0.01	0.075
	Paper-based questionnaire vs. Google Forms	0.45	0.19 to 0.70	0.001

The defensive-practice model had R2 = 0.066 and adjusted R2 = 0.037 and was fitted on 170 respondents. The legal-awareness model had R2 = 0.077 and adjusted R2 = 0.049 and was fitted on 172 respondents; professional clinical experience was entered as a binary indicator. CI: confidence interval.

## Data Availability

The data presented in this study are available on request from the corresponding authors due to privacy and ethical considerations related to participant-level questionnaire data.

## Supplementary Materials

The following supporting information can be downloaded at https://www.mdpi.com/article/10.3390/dj14090579/s1, File S1: English Version of the Questionnaire Used in the Study.

## Abbreviations

The following abbreviations are used in this manuscript:

CI	Confidence interval
CME	Continuing medical education
FDR	False discovery rate
IQR	Interquartile range
OR	Odds ratio
SD	Standard deviation
STROBE	Strengthening the Reporting of Observational Studies in Epidemiology
WHO	World Health Organization

## References

[1] World Health Organization (2021). Global Patient Safety Action Plan 2021–2030: Towards Eliminating Avoidable Harm in Health Care.

[2] Miziara I.D., Miziara C.S.M.G. (2022). Medical Errors, Medical Negligence and Defensive Medicine: A Narrative Review. Clinics.

[3] Ries N.M., Jansen J. (2021). Physicians’ Views and Experiences of Defensive Medicine: An International Review of Empirical Research. Health Policy.

[4] Pischedda G., Marinò L., Corsi K. (2023). Defensive Medicine through the Lens of the Managerial Perspective: A Literature Review. BMC Health Serv. Res..

[5] Pallocci M., Treglia M., Passalacqua P., Tittarelli R., Zanovello C., De Luca L., Caparrelli V., De Luna V., Cisterna A.M., Quintavalle G. (2023). Informed Consent: Legal Obligation or Cornerstone of the Care Relationship?. Int. J. Environ. Res. Public Health.

[6] Bolcato V., Fassina G., Rodriguez D., Russo M., Aprile A. (2024). Comparative Study on Informed Consent Regulation in Health Care among Italy, France, United Kingdom, Nordic Countries, Germany and Spain. J. Forensic Leg. Med..

[7] Arbel E., Reese A., Oh K., Mishra A. (2024). Medical Law and Medical School Curricula: A Systematic Review. Cureus.

[8] Johnston W.F., Rodriguez R.M., Suarez D., Fortman J. (2013). Study of Medical Students’ Malpractice Fear and Defensive Medicine: A “Hidden Curriculum”?. West. J. Emerg. Med..

[9] Arafa A., Negida A., Elsheikh M., Emadeldin M., Hegazi H., Senosy S. (2023). Defensive Medicine Practices as a Result of Malpractice Claims and Workplace Physical Violence: A Cross-Sectional Study from Egypt. Sci. Rep..

[10] Can Özdemir R., Işık M.T., Aslan A., Ayaz M. (2024). Factors Affecting Physician Fear of Malpractice and Defensive Medicine Practices: A Cross-Sectional Study. J. Acad. Res. Med..

[11] Plaiasu M.C., Alexandru D.O., Nanu C.A. (2022). Physicians’ Legal Knowledge of Informed Consent and Confidentiality. A Cross-Sectional Study. BMC Med. Ethics.

[12] von Elm E., Altman D.G., Egger M., Pocock S.J., Gøtzsche P.C., Vandenbroucke J.P., STROBE Initiative (2007). The Strengthening the Reporting of Observational Studies in Epidemiology Statement: Guidelines for Reporting Observational Studies. PLoS Med..

[13] Nikkhahmanesh N., Kang P., vanSonnenberg E. (2023). Evaluating Medical Students’ Knowledge of Medical Malpractice: A Pilot Study. Int. J. Med. Stud..

[14] Bhadauria U.S., Dasar P.L., Sandesh N., Mishra P., Godha S. (2018). Medico-Legal Aspect of Dental Practice. Med. Pharm. Rep..

[15] Jameson L.M., Al-Tarawneh S.K. (2022). Informed Consent from a Historical, Societal, Ethical, Legal, and Practical Perspective. J. Prosthodont..

[16] Devadiga A. (2014). What’s the Deal with Dental Records for Practicing Dentists? Importance in General and Forensic Dentistry. J. Forensic Dent. Sci..

[17] Hamasaki T., Kato H., Kumagai T., Hagihara A. (2021). Dentists’ Legal Liability and Duty of Explanation in Dental Malpractice Litigation in Japan. Int. Dent. J..

[18] Al-Azri N.H. (2020). Providing Legal Education for Medical Students in Arab Gulf Cooperation Council Countries. J. Med. Educ. Curric. Dev..

[19] Chen W.T., Fu C.P., Chang Y.D., Shiao Y.C., Chen P.Y., Wang C.C. (2022). Developing an Innovative Medical Ethics and Law Curriculum—Constructing a Situation-Based, Interdisciplinary, Court-Based Learning Course: A Mixed Methods Study. BMC Med. Educ..

[20] Baungaard N., Skovvang P.L., Assing Hvidt E., Gerbild H., Andersen M.K., Lykkegaard J. (2022). How Defensive Medicine Is Defined in European Medical Literature: A Systematic Review. BMJ Open.

[21] Zheng J., Lu Y., Li W., Zhu B., Yang F., Shen J. (2023). Prevalence and Determinants of Defensive Medicine among Physicians: A Systematic Review and Meta-Analysis. Int. J. Qual. Health Care.

[22] Misir S.E., Uğrak U., Topsakal K.G., Dalgali P., Duran G.S., Görgülü S. (2025). Evaluation of Dentists’ Malpractice Fears and Defensive Dentistry Attitudes: A Scale Development Study. BMC Oral Health.

[23] Lorenc T., Khouja C., Harden M., Fulbright H., Thomas J. (2024). Defensive Healthcare Practice: Systematic Review of Qualitative Evidence. BMJ Open.

[24] Eftekhari M.H., Parsapoor A., Ahmadi A., Yavari N., Larijani B., Shamsi Gooshki E. (2023). Exploring Defensive Medicine: Examples, Underlying and Contextual Factors, and Potential Strategies—A Qualitative Study. BMC Med. Ethics.

[25] Goetz K., Oldenburg D., Strobel C.J., Steinhäuser J. (2024). The Influence of Fears of Perceived Legal Consequences on General Practitioners’ Practice in Relation to Defensive Medicine—A Cross-Sectional Survey in Germany. BMC Prim. Care.

[26] Bruers J.J.M., van Dam B.A.F.M., Gorter R.C., Eijkman M.A.J. (2016). The Impact of a Formal Complaint on Dutch Dentists’ Professional Practice: A Survey Study. BMC Oral Health.

[27] Klemann D., Mertens H., van Merode F. (2022). Trends and Developments in Medical Liability Claims in The Netherlands. Healthcare.

[28] Abomalik A.M., Alsubaie S.A., Alqahtani A.S., Alqahtani F.M., Alqahtani N.M., Alshahrani M.S. (2022). A Retrospective Assessment of the Dental Malpractice Cases in Riyadh, Saudi Arabia. J. Pharm. Bioallied Sci..

[29] Mello M.M., Frakes M.D., Blumenkranz E., Studdert D.M. (2020). Malpractice Liability and Health Care Quality: A Review. JAMA.

[30] Costanza-Gugiu F., Cernega A., Pițuru S.-M. (2025). Medico-Legal Implications and Risk Management Strategies in Orthodontic Practice: An Analytical Literature Review. Healthcare.

