# Supplementary material for: Title Medico-Legal Preparedness in Dental Education: Training Gaps, Defensive Practice and Curriculum Implications Among Romanian Dental Students and Dentists
Source: Dent J (Basel). 2026 Sep 9;14(9):579. doi: 10.3390/dj14090579 (PMC13605883; doi:10.3390/dj14090579)
Supplement: Supplementary file 1 [file dentistry-14-00579-s001.zip › dentistry-4500216-supplementary.pdf]

# Supplementary File S1. English Version of the Questionnaire Used in the Study

Study title: Medico-Legal Preparedness in Dental Education: Training Gaps, Defensive Practice and Curriculum Implications Among Romanian Dental Students and Dentists

## General note

This supplementary file presents the English version of the questionnaire administered in Romanian for this cross-sectional study. The instrument was developed by the research team and had not been used or externally validated previously. Before full administration, it underwent a preliminary comprehensibility test with 10 eligible participants; feedback led only to minor wording and presentation refinements that did not change item intent, response options or scoring, and these responses were retained in the analytical dataset.

The instrument was designed to assess objective legal-knowledge items, perceived medico-legal preparedness, awareness, attitudes and reported or anticipated defensive-practice tendencies among early-year and clinical-year dental students, dental residents and practising dentists. For respondents without independent clinical responsibility, defensive-practice items represent anticipated responses rather than established clinical behaviour. The English version is provided for transparency, reproducibility and peer review.

Unless otherwise stated, participants were asked to select one answer. Multiple-response items are explicitly marked.

## Questionnaire title

**Perceptions of Dental Malpractice and Defensive Practice in Romania**

## Introductory statement provided to respondents

Please answer the following questions regarding dental malpractice and defensive practice in Romania.

The data collected will be confidential and will be used only in aggregated form for statistical analysis and scientific reporting.

The study is addressed to dentists and students enrolled in dental medicine programmes.

## Section 1. Respondent Characteristics

**1. Age group**

*Select one answer.*

- 18–23 years
- 24–30 years
- 31–40 years
- 41–50 years
- Over 50 years

**2. Gender**

*Select one answer.*

- Female
- Male
- Prefer not to answer

**3. Current professional status**

*Select one answer.*

- Dental student — Year I
- Dental student — Year II
- Dental student — Year III
- Dental student — Year IV
- Dental student — Year V
- Dental student — Year VI
- Dental resident
- Specialist dentist
- Senior-grade dentist (Romanian professional grade: medic dentist primar)

**4. Main environment of activity or training**

*Select one answer.*

- University setting (university clinics/faculty)
- Public sector (public hospital or outpatient clinic)
- Private sector (private dental office or clinic)
- Mixed
- No clinical activity yet (early educational stage)

**5. Professional experience in clinical dental practice**

*Select one answer.*

- No clinical activity / clinical placements yet (early educational stage)
- University clinical placements only
- Less than 1 year of independent practice
- 1–5 years
- 5–10 years
- More than 10 years

**6. Have you attended courses or training sessions on medical legislation or malpractice?**

*Select one answer.*

- Yes, as part of the university curriculum
- Yes, through continuing medical education
- Yes, both as part of the university curriculum and through continuing medical education
- No

## Section 2. Legal Knowledge Regarding Malpractice

**7. Are you familiar with the concept of dental malpractice?**

*Select one answer.*

- Yes
- No

- I have heard of the term, but I do not know the details

**8. Medical malpractice in Romania is regulated by:**

*Select one answer.*

- Law no. 95/2006 on healthcare reform
- The Criminal Code
- Law no. 46/2003 on patients' rights
- I do not know

**9. Which of the following elements must be cumulatively proven in order to establish malpractice?**

*Select all correct answers.*

- The existence of a medical act, including prevention, diagnosis or treatment
- The occurrence of damage to the patient, either material or moral
- Fault attributable to the dentist
- A causal link between the act and the damage
- The dentist's deliberate intention to cause harm
- I do not know

**10. Which of the following institutions or organisations may be involved in managing a case of dental malpractice?**

*Select one or more answers.*

- Civil or criminal court
- Romanian College of Dentists
- Monitoring and Professional Competence Commission for Malpractice Cases
- Insurance company
- Public Health Directorate
- Ministry of Health
- I do not know

**11. Which of the following forms of liability may be engaged in a case of dental malpractice?**

*Select one or more answers.*

- Civil liability, when the patient seeks compensation
- Criminal liability, when the dentist's act constitutes a criminal offence
- Disciplinary liability, when the dentist has committed a disciplinary breach
- Administrative liability
- I do not know

**12. Which of the following situations do you consider may represent dental malpractice?**

*Select one or more answers.*

- Incorrect diagnosis
- Incorrect treatment
- Lack of informed consent
- Complications not explained to the patient
- Violation of a patient's right
- I do not know

**13. From which sources have you developed your legal knowledge related to malpractice?**

*Select all that apply.*

- University curriculum, including courses and seminars
- Continuing medical education
- Specialist literature or indexed articles
- Case law or court decisions
- Mass media, internet or social networks
- Colleagues, mentors or specialists
- Direct personal experience, including involvement in a case
- I do not have clear sources of legal knowledge

## Section 3. Perceptions Regarding the Malpractice Phenomenon

**14. In your opinion, dental malpractice in Romania is:**

*Select one answer.*

- Very frequent
- Frequent
- Moderate
- Rare
- I do not know / I cannot assess

**15. Compared with 5–10 years ago, the number of dental malpractice complaints in Romania has:**

*Select one answer.*

- Increased significantly
- Increased slightly
- Remained constant
- Decreased
- I do not know

**16. In your opinion, what are the main causes of dental malpractice?**

*Select up to three answers.*

- Insufficient professional training
- Lack of clinical experience
- Deficient dentist–patient communication
- Absent or inadequate informed consent
- Dentist overload / insufficient time per patient
- Inadequate technical resources
- Professional negligence
- Unrealistic patient expectations
- Insufficient knowledge of legislation by the dentist

**17. Have you personally been involved in, or do you know a colleague involved in, a malpractice situation or complaint?**

*Select one answer.*

- Yes, personally
- Yes, a colleague, but not myself

- Both
- No
- Prefer not to answer

**18. Do you consider that patients in Romania tend to report malpractice more frequently than they did 5 years ago?**

*Select one answer.*

- Yes, significantly more frequently
- Yes, slightly more frequently
- Approximately the same
- No, less frequently
- I do not know

**19. How prepared do you consider yourself to manage a malpractice case or complaint?**

*Select one answer.*

- Not prepared at all
- Slightly prepared
- Moderately prepared
- Well prepared
- Very well prepared

**20. Do you consider that university training or residency has sufficiently prepared you for malpractice prevention?**

*Select one answer.*

- Yes, completely
- Partially
- No
- I do not know

**21. Which measures do you consider effective for reducing the risk of malpractice?**

*Select up to three answers.*

- Mandatory continuing medical education
- Standardised clinical protocols
- Rigorous documentation of medical acts
- Effective and empathetic communication with the patient
- Mandatory courses in medical legislation within the curriculum
- Properly adjusted malpractice insurance
- Closer supervision of early-career dentists
- Reform of the current legal framework

**22. Do you consider that the current system effectively manages dental malpractice cases in Romania?**

*Select one answer.*

- Yes
- Partially
- No
- I do not know

**23. How much time do you consider should be allocated to learning the legislation specific to your field of activity?**

*Select one answer.*

- None
- Less than 1 hour per month
- 1–3 hours per month
- 4–6 hours per month
- More than 6 hours per month
- I consider that legal education should be continuous

**24. In the event of a presumed malpractice act, as a dentist, you would choose to:**

*Select one answer.*

- Immediately inform the patient and offer remediation
- Consult a specialist colleague before informing the patient
- Consult a legal professional or the insurer
- Remedy the situation without explicitly informing the patient
- I do not know / I have not thought about this

## Section 4. Perceptions Regarding Fear of Malpractice and Reported or Anticipated Defensive Practice

For the following statements, please indicate your level of agreement or intensity using the scale below. Items phrased as 'have' or 'would' allow respondents without independent clinical practice to report anticipated tendencies.

| 1<br>Not at all | 2<br>Slightly | 3<br>Moderately | 4<br>To a great extent | 5<br>To a very great extent |
|---|---|---|---|---|

### A. Medico-Legal Fear

**25. I fear that a patient could file a malpractice complaint against me even if I have followed the protocols.**

**26. I am concerned about the possibility of being the subject of a civil lawsuit for a medical act.**

**27. The thought of a potential complaint causes me anxiety in my daily activity.**

**28. I fear that the legal system does not sufficiently understand the specific nature of dental practice.**

**29. Fear of malpractice affects my satisfaction with the profession.**

### B. Defensive-Practice Tendencies and Clinical Behaviours

**30. Fear of malpractice makes me more cautious when making clinical decisions.**

**31. I have avoided, or would avoid, complex clinical cases because of legal risk.**

**32. Fear of malpractice influences the way I communicate with patients.**

**33. I prefer to refer the patient to another specialist in order to avoid a potential risk.**

**34. I frequently think about legal consequences while performing clinical procedures.**

### C. Defensive-Practice Behaviours

**35. I recommend additional paraclinical investigations beyond what is strictly clinically necessary, for legal protection.**

**36. I request extremely detailed informed consent even for routine procedures.**

**37. I document the medical act more extensively than clinically necessary in order to protect myself.**

**38. I refer patients to specialists in situations that I could manage myself, out of legal caution.**

**39. I extend post-treatment monitoring beyond what is clinically necessary, out of legal caution.**

**40. I prescribe additional medication, such as prophylactic antibiotics, mainly for legal protection.**

### D. Clinical Avoidance and Decisions Influenced by Legal Risk

**41. I avoid high-risk procedures, such as difficult extractions or complex implants, because of legal risk.**

**42. I refuse, or would refuse, patients with expectations perceived as unrealistic in order to avoid complaints.**

**43. I prefer more conservative treatments, even if they are not the most clinically effective, for legal safety.**

**44. I have recommended, or would recommend, a simpler treatment plan than the clinically optimal one.**

**45. I avoid expressing diagnostic uncertainty to the patient because I fear it could be interpreted negatively.**

**46. I have omitted, or would omit, informing the patient about a minor complication in order to avoid a complaint.**

### E. Perceptions Regarding Medical Legislation and Legal Education

**47. Good knowledge of medical legislation significantly reduces the risk of malpractice.**

**48. The current legal framework in Romania sufficiently protects the dentist.**

**49. The current legal framework in Romania sufficiently protects the patient.**

**50. There are significant discrepancies between the current legislation and the reality of dental practice.**

**51. Knowledge of legislation gives me greater confidence in managing malpractice risk.**

**52. Dental students should study medical legislation in greater depth.**

**53. Medical legislation courses in dental school are sufficient for dental practice.**

**54. Dentists should collaborate more closely with legal professionals specialised in the field.**

## Scoring of Exploratory Composite Scores

Likert-type items were scored from 1 to 5, with higher values indicating greater intensity of the corresponding construct. Each exploratory composite index was calculated as the arithmetic mean of its component items when at least 80% of the relevant responses were available. The indices were analysed as continuous exploratory measures; no externally validated calibration, diagnostic threshold or categorical cut-off was applied. Cronbach's alpha was used to describe internal consistency only and does not establish validity or unidimensionality.

- Medico-legal fear score: mean of items 25–29.

- Defensive-practice tendency score: mean of all 17 items numbered 30–46.
- Legal awareness score: mean of items 47, 48, 49, 51, 52 and 54.

Because the questionnaire was author-developed and newly created, and no formal content-validity-index assessment, exploratory factor analysis, external validation or test–retest assessment was undertaken, these indices should be interpreted as exploratory composite measures rather than validated psychometric scales.
